# Ring-like late gadolinium enhancement pattern in antimalarial-induced cardiomyopathy

**DOI:** 10.1093/ehjcr/ytag276

**Published:** 2026-04-17

**Authors:** Lévi-Dan Azoulay, Alexis Mathian, Nadjia Kachenoura, Zahir Amoura, Alban Redheuil

**Affiliations:** Sorbonne Université, Laboratoire d'Imagerie Biomédicale, Inserm, CNRS, 15 Rue de l'École de Médecine, Paris 75006, France; Sorbonne Université, Assistance Publique-Hôpitaux de Paris, Groupement Hospitalier Pitié-Salpêtrière (GHPS), Centre de Référence des maladies auto-immunes et auto-inflammatoires systémiques rares de l'adulte d'Ile-de-France, Centre et Martinique, Service de Médecine Interne 2, Institut E3M, Inserm UMRS, Centre d’Immunologie et des Maladies Infectieuses (CIMI-Paris), 47-83 Boulevard de l’hôpital, Paris 75013, France; Sorbonne Université, Assistance Publique-Hôpitaux de Paris, Groupement Hospitalier Pitié-Salpêtrière (GHPS), Centre de Référence des maladies auto-immunes et auto-inflammatoires systémiques rares de l'adulte d'Ile-de-France, Centre et Martinique, Service de Médecine Interne 2, Institut E3M, Inserm UMRS, Centre d’Immunologie et des Maladies Infectieuses (CIMI-Paris), 47-83 Boulevard de l’hôpital, Paris 75013, France; Sorbonne Université, Laboratoire d'Imagerie Biomédicale, Inserm, CNRS, 15 Rue de l'École de Médecine, Paris 75006, France; Sorbonne Université, Assistance Publique-Hôpitaux de Paris, Groupement Hospitalier Pitié-Salpêtrière (GHPS), Centre de Référence des maladies auto-immunes et auto-inflammatoires systémiques rares de l'adulte d'Ile-de-France, Centre et Martinique, Service de Médecine Interne 2, Institut E3M, Inserm UMRS, Centre d’Immunologie et des Maladies Infectieuses (CIMI-Paris), 47-83 Boulevard de l’hôpital, Paris 75013, France; Sorbonne Université, Laboratoire d'Imagerie Biomédicale, Inserm, CNRS, 15 Rue de l'École de Médecine, Paris 75006, France; Sorbonne Université, Assistance Publique-Hôpitaux de Paris, Unité d’Imagerie Cardiovasculaire et Thoracique (ICT), Institut de Cardiologie, Groupement Hospitalier Pitié-Salpêtrière (GHPS), 47-83 Boulevard de l’hôpital, Paris 75013, France; Sorbonne Université, Assistance Publique-Hôpitaux de Paris, Equipe Recherche DMU DIAMENT, 47-83 Boulevard de l’hôpital, Paris 75013, France; Sorbonne Université, Assistance Publique-Hôpitaux de Paris, Institut de Cardiométabolisme et de Nutrition (ICAN), 47-83 Boulevard de l'hôpital, Paris 75013, France

**Keywords:** Systemic lupus erythematosus, Cardiac magnetic resonance, Antimalarial-induced cardiomyopathy

A 67-year-old woman with systemic lupus erythematosus (SLE) and 22 years of hydroxychloroquine therapy presented with NYHA III–IV dyspnoea, markedly elevated NT-proBNP and troponin T, and fragmented QRS with Q waves and ST-segment elevation. Cardiac magnetic resonance (CMR) showed left ventricular hypertrophy, reduced left ventricular ejection fraction (32%) and extensive midwall intramyocardial late gadolinium enhancement (LGE) forming a circumferential ring-like pattern (*Figure 1*, panels A–C).

A 60-year-old woman with SLE and 31 years of hydroxychloroquine exposure presented with NYHA IV dyspnoea, high NT-proBNP and troponin T levels, and fragmented QRS. CMR demonstrated left ventricular hypertrophy, mildly reduced systolic function (50%) and extensive midwall ring-like LGE (*Figure 1*, panels E–G).

A 65-year-old woman with SLE treated with hydroxychloroquine for 43 years presented with chest pain, cardiac biomarkers elevation, and ventricular ectopy. CMR revealed concentric hypertrophy, preserved ejection fraction (60%), mild pericardial effusion, and ring-like LGE (*Figure 1*, panels I–K).

All three patients had unobstructed coronary arteries and underwent endomyocardial biopsy which results were consistent with antimalarial-induced cardiomyopathy, including diffuse myocyte vacuolization, cytoplasmic inclusions, interstitial fibrosis, and curvilinear bodies on electron microscopy (*Figure 1*, panels D, H, and L). Further details are provided in Supplementary material online.

**Figure 1:**
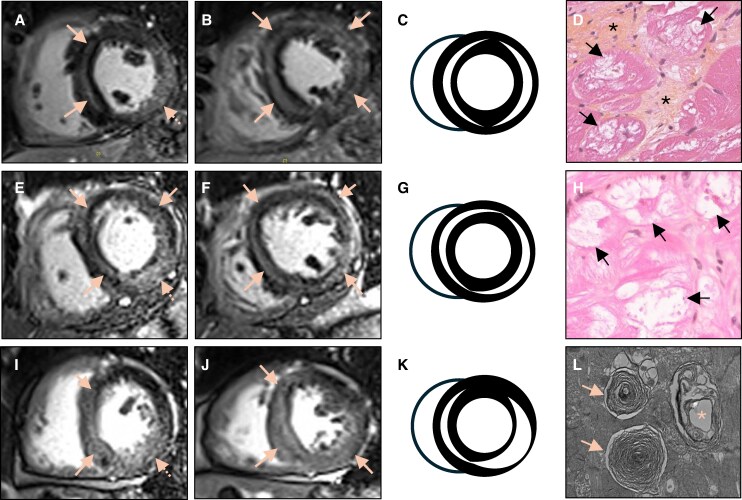
*(A–C)* Short-axis LGE images from patient #1 demonstrating extensive midwall enhancement forming a complete ring-like configuration with marked lateral and septal walls involvement. The dotted arrow corresponds to a nearly transmural basal inferolateral enhancement. *(D)* Corresponding endomyocardial biopsy (HES staining) showing vacuolization and myocytes ballooning (arrows), intracytoplasmic inclusions, and fibrosis (asterisk). *(E–G)* Short-axis LGE views from patient #2 reveal a diffuse midwall enhancement pattern, involving basal, mid-ventricular, and apical anteroseptal, inferoseptal, and lateral segments, reconstituting a circumferential ring-like aspect. The dotted arrow shows a nearly transmural basal inferolateral enhancement. *(H)* Corresponding endomyocardial biopsy (HES staining) showing vacuolization and myocytes ballooning with intracyoplasmic inclusions (arrows). *(I–K)* Short-axis LGE images from patient #3 show marked inferior and inferolateral nearly transmural enhancement combined with midwall septal anterior and inferior involvement, together producing a ring-like pattern. The dotted arrow shows a nearly transmural basal inferolateral enhancement. A mild pericardial effusion can also be seen. *(L)* Corresponding endomyocardial biopsy (electron microscopy) showing lamellar myeloid bodies (arrows) and a mitochondrion with disorganized cristae (asterisk).

Across all three cases, CMR consistently disclosed a distinctive midwall ring-like LGE phenotype. This pattern, classically associated with inherited arrhythmogenic cardiomyopathies, has not previously been described in antimalarial-induced cardiomyopathy.^1,2^ Our observations suggest that hydroxychloroquine cardiotoxicity may converge towards a ring-like myocardial scar phenotype, representing a novel imaging signature of advanced disease.

## Supplementary Material

ytag276_Supplementary_Data

## Data Availability

The de-identified data are available in Supplementary material Online.
