# Supplementary material for: Ring-like late gadolinium enhancement pattern in antimalarial-induced cardiomyopathy
Source: Eur Heart J Case Rep. 2026 Apr 17;10(5):ytag276. doi: 10.1093/ehjcr/ytag276 (PMC13165417; doi:10.1093/ehjcr/ytag276)
Supplement: ytag276_Supplementary_Data [file ytag276_supplementary_data.pdf]

Supplementary Material

This appendix has been provided by the authors to give readers additional information about their work.

Supplement to: Azoulay L-D et al., "Ring-Like Late Gadolinium Enhancement Pattern in Antimalarial-induced Cardiomyopathy"

Supplementary Table S1. Characteristics of patients with biopsy-proven antimalarial-induced cardiomyopathy.

	**Patient #1**	**Patient #2**	**Patient #3**
**Clinical features**			
Age at CMR, years	67	60	65
HCQ exposure, years	22	31	43
Symptoms	HFrEF<br>Dyspnea<br>NYHA IIII	HFpEF<br>Dyspnea<br>NYHA IV	Chest pain<br>Dyspnea<br>NYHA II
Electrocardiogram	Fragmented QRS,<br>Q waves,<br>ST-segment elevation	Fragmented QRS	Premature ventricular contractions
**Biological features**			
NT-proBNP pg/mL (N<300)	22,278	35,000	2,378
Troponin T, ng/L (N<14)	587	746	200
CPK, mg/L (N<190)	250	208	269
**Exclusion of CAD**	Yes (CTCA)	Yes (CA)	Yes (CA)
**Imaging features**			
LVEF	32	50	60
LVH	Yes	Yes	Yes
LVM/LVEDV	1,5	1,4	1,4
GLS, %	-9	-12	-
LGE	Yes	Yes	Yes
LGE midwall pattern	Yes	Yes	Yes
LGE extent	35%	24%	29%
Native T1, ms (N<1077)	1102	924	1165
ECV, % (N<25)	30	33	35
T2, ms (N<53)	56	58	56
**Positive EMB**	Yes	Yes	Yes

Abbreviations: CA, Coronary Angiogram; CAD, Coronary Artery Disease; CMR, Cardiac Magnetic Resonance; CPK, Creatine Phosphokinase; CTCA, Computed Tomography Coronary Angiography; ECG, Electrocardiogram; ECV, Extracellular Volume fraction; EMB, Endomyocardial Biopsy; GLS, Global Longitudinal Strain; HCQ, Hydroxychloroquine; HFrEF, Heart Failure with reduced Ejection Fraction; HFpEF, Heart Failure with preserved Ejection Fraction; LGE, Late Gadolinium Enhancement; LVEF, Left Ventricular Ejection Fraction; LVH, Left Ventricular Hypertrophy; LVM/LVEDV, Left Ventricular Mass / Left Ventricular End-Diastolic Volume ratio; NT-proBNP, N-terminal pro–B-type natriuretic peptide; NYHA, New York Heart Association functional classification; T1, Native T1 relaxation time; T2, T2 relaxation time.

Supplementary Figure S1. Electrocardiogram features in included patients with antimalarial-induced cardiomyopathy.

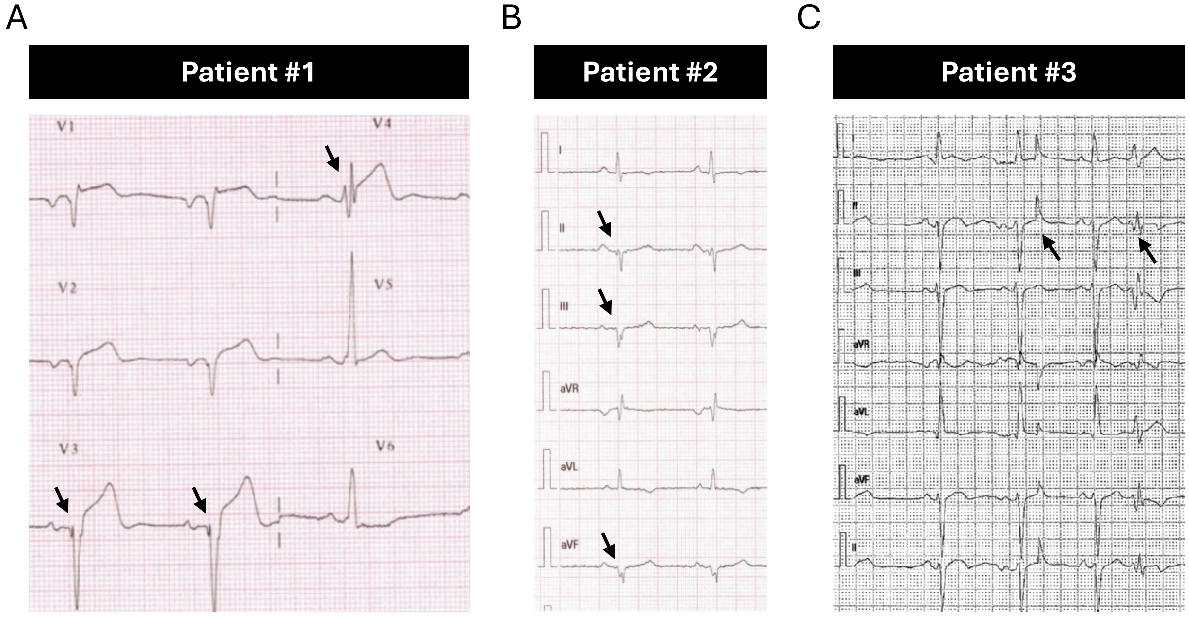

A. Fragmented QRS complexes (arrows) with anterior Q waves and ST-segment elevation in V1–V3, consistent with advanced structural and electrical remodeling.
B. Fragmented and microvolted QRS complexes (arrows) in leads DII, DIII, and aVF, reflecting electrical remodeling in the inferior territory.
C. Electrocardiogram demonstrates frequent premature ventricular complexes (arrows) with variable morphology.

Supplementary Figure S2. Key features of antimalarial-induced cardiomyopathy on cardiac magnetic resonance imaging.

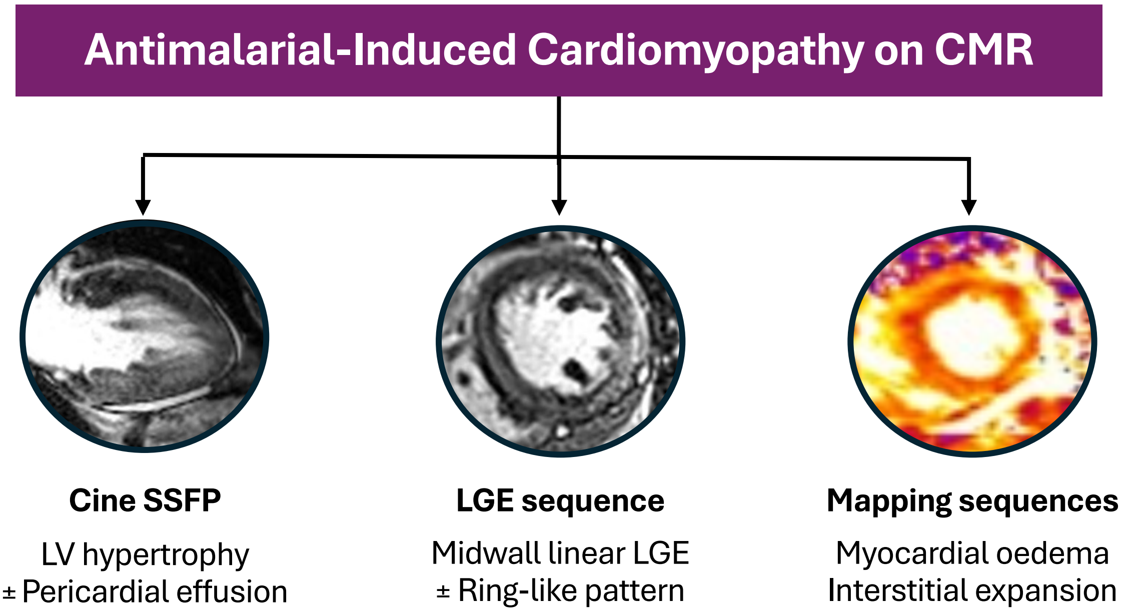

T2 relaxation time was elevated in all three cases and indicated mild myocardial oedema. Extra-cellular volume was consistently elevated, suggesting interstitial expansion.

Abbreviations: CMR, cardiac magnetic resonance; LGE, late gadolinium enhancement; LV, left ventricular, SSFP, steady-state free precession.
